# Cervical spine involvement in patients with juvenile idiopathic arthritis - MRI follow-up study

**DOI:** 10.1186/1546-0096-12-9

**Published:** 2014-03-04

**Authors:** Toni Hospach, Jan Maier, Peter Müller-Abt, Anita Patel, Gerd Horneff, Thekla von Kalle

**Affiliations:** 1 Division of Pediatric Rheumatology, Children's Hospital, Olgahospital Stuttgart, teaching hospital of the University of Tuebingen, Bismarckstr. 8, 70176 Stuttgart, Germany; 2 Radiologic Institute, Children's Hospital, Olgahospital Stuttgart, teaching hospital of the University of Tuebingen, Bismarckstr. 8, 70176 Stuttgart, Germany; 3 Children's Hospital, Asklepios Klinik, Arnold-Janssenstr. 29, 53757 Sankt Augustin, Germany

## Abstract

**Background:**

To describe MRI and clinical findings in patients with juvenile idiopathic arthritis with cervical spine involvement at onset and follow-up under therapy.

**Methods:**

13 patients with signs of cervical spine involvement in juvenile idiopathic arthritis with a median disease duration of 1.7 years were included in the study. Clinical records and MR images were retrospectively analyzed according to symptoms and findings concerning the cervical spine.

**Results:**

At the onset of cervical spine involvement all patients showed limited range of motion, whereas only 5 of them complained of pain. In MR images joint hyperintensity, contrast enhancement, malalignment, ankylosis, erosion and narrowing of the spinal canal at cranio-cervical junction were found at 28, 32, 15, 2, 2 and 3 sites in 12 (93%), 13 (100%), 8 (62%), 2 (15%), 2 and 3 (20%) patients respectively. 3 of the 5 patients with pain (60%) showed ankylosis, erosions or narrowing of the spinal canal at cranio-cervical junction on MRI. At follow-up - after a median disease duration of cervical spine arthritis of 2.1 years and a variable duration of treatment with methotrexate (all patients) and biological agents (12 patients) - joint hyperintensity, enhancement and malalignment decreased to 15, 19 and 6 sites in 10 (77%), 11 (85%) and 3 (20%) patients respectively whereas ankylosis, erosion and narrowing of the spinal canal at cranio-cervical junction increased to 7, 6 and 4 sites in 3 (20%), 4 (31%) and 4 patients respectively. Pain was no longer reported, but 9 of 13 (69%) patients still had a limited range of motion with 6 of them (46%) showing skeletal changes on MRI.

**Conclusions:**

This first MRI based follow-up study shows that cervical spine arthritis can follow a severe disease course in juvenile arthritis. While malalignments and inflammation sites decreased osseous changes with erosions, ankylosis, and narrowing of the spinal canal increased under treatment despite only minor subjective complaints. Therefore close MRI monitoring of these patients appears to be reasonable.

## Background

In contrast to adult rheumatoid arthritis, where numerous studies have shown a high prevalence of involvement of the cervical spine, few studies have been published examining this entity in juvenile idiopathic arthritis (JIA) [1-8]. Nearly all of the studies have used conventional radiographs as the diagnostic standard [9-13]. In a comparative study [14] magnetic-resonance imagining (MRI) showed a higher sensitivity than plain radiographs and computed tomography in diagnosing cervical spine arthritis, but reports on cross-sectional MRI findings in JIA with cervical spine involvement are rare [15,16]. Follow-up studies combining clinical and MRI findings are not reported for cervical spine arthritis. Only one recent study followed clinical disease activity and MRI findings of knee involvement in JIA [17] and in conclusion recommended MRI as a responsive outcome measure to evaluate disease activity.

The aim of our retrospective study was to describe and compare MRI and clinical findings at the onset and follow-up of cervical spine arthritis in JIA patients.

## Methods

We searched our clinical records and radiological data base for patients with cervical spine arthritis in JIA and included patients who had both clinical and MRI examination at the onset of signs or symptoms of cervical spine arthritis (limited range of motion and/or pain) and at follow-up. Our search criteria were limited to individuals who had follow-up examinations between 1.1.2010 and 31.12.2011. All MR examinations were performed with a 12-channel head coil and a dedicated neck coil at 1.5 T (Magnetom Avanto Siemens Healthcare, Erlangen, Germany). Sequences were 3 mm sagittal and coronal short Tau inversion recovery (STIR) and T1 fat saturated dynamic imaging after injection of contrast medium with 30 sec. temporal resolution followed by a 3D T1 sequence with water excitation (fat suppression technique) in 1 mm sagittal partitions and high in-plane resolution; image parameters see Table 1. Two pediatric radiologists with more than 10 years experience independently assessed MR images of the cranio-cervical junction and the cervical spine for abnormally high signal intensity of joints, bone marrow and soft tissue before and after contrast enhancement. Form, contour and alignment of the cranio-cervical junction and the cervical spine were evaluated to detect deformities, ankyloses and malpositions. All patients and their parents gave written informed consent to the examination. The retrospective evaluation of anonymized data is in accordance with the regulations of the local ethics committee. No magnetic resonance images were acquired for the study purposes.

**Table 1:** Image parameters

**Sequence**	**Contrast material**	**TR (msec)**	**TE (msec)**	**TI (msec)**	**Voxel size (mm)**	**Coil**	**Other parameters**
STIR 2D coronal	Pre-contrast	5300-6200	60	135	0.4-0.8 × 0.6-1.2 × 3.0	Head coil	
STIR 2D sagittal	Pre-contrast	3900	60	135	0.4 × 0.4 × 3.0	Ring coil	
T1 gradient echo 3D VIBE axial	Contrast-enhanced Dynamic scanning	5.0	1.4	-	0.8 × 0.6 × 2.0	Head coil	Fat saturation. Duration 0.5 min. k-space-center at 0.16 min.
T1 gradient echo 3D sagittal	Post-contrast	400-630	11-20	-	0.5 × 0.5 × 1.0	Head coil	Water excitation

Proposals for measurement of atlanto-axial dislocations had been derived from plain radiographs in adults and were not applicable to our cohort [18,19]. We therefore matched images of children who had been examined for reasons other than diseases with joint or spine involvement for age and used them as normal controls.

**Figure 1:**
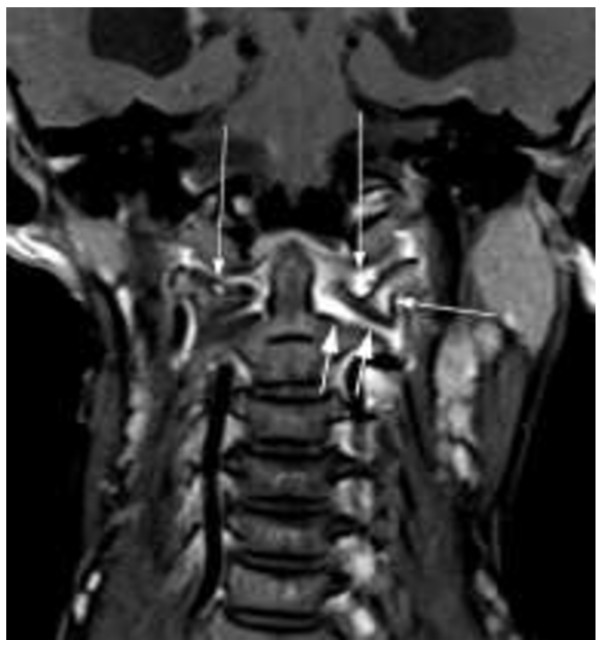
**9-year-old girl with JIA at first diagnosis of cervical arthritis (3 mm coronal T1 spinecho sequence with fat saturation).** Malalignment of occiput, atlas and axis. Contrast enhancement of atlanto-occipital joints with a small erosion of the right and a large erosion of the left lateral mass of the atlas (long arrows). Contrast enhancement of the bone marrow in the atlas (short arrow). Contrast enhancement of the widened left atlanto-axial joint (two short arrows).

**Figure 2:**
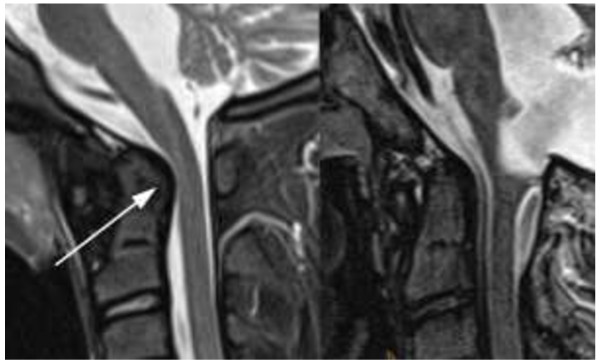
**Left: 13-year-old girl with JIA. STIR 3 mm sagittal.** Enlarged dens with bulging dorsal contour (arrow) and narrowing of the spinal canal at the cranio-cervical junction (arrowheads). Right: Normal control. 13-year-old boy without JIA and normal size of his dens.

**Figure 3:**
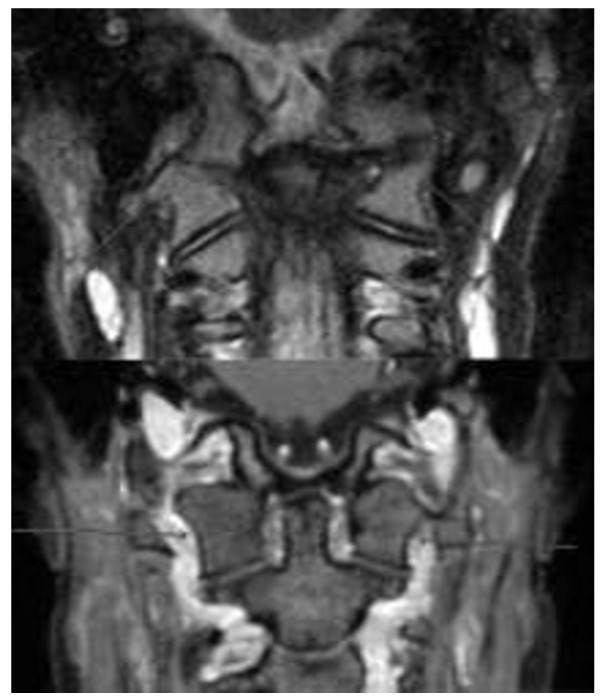
**Two patients with ankylosis.** Above: 9-year-old boy (3 mm coronal STIR). Ankylosis with partial fusion of the occipital condyle and atlas on the right with a blurring of the osseous contours on the left. Below: 17-year-old girl with bilateral complete fusion of occipital condyle and atlas (1 mm coronal reconstruction from high-resolution 3D T1 gradient echo post-contrast).

We defined malalignment as any abnormal (or partially abnormal) position of articular surfaces of atlanto-occipital, atlanto-axial or cervical facet joints, or of two vertebrae with respect to each other (Figure 1). This definition included joint subluxation. We defined ankylosis as osseous bridges between two or more bones which are normally separate (Figure 2). An erosion is an interruption of the osseous joint surface with signs of inflammation such as a hyperintense signal in STIR images before and in T1 images after contrast injection (Figure 1). Narrowing of the spinal canal at the cranio-cervical junction caused by hypertrophy of the dens was assessed by comparing the sagittal diameter of the spinal canal at the cranio-cervical junction to its diameter in the upper cervical spine and then to the reference image (Figure 3). We considered contrast enhancement of a joint space as sign of arthritis (synovial hypertrophy and/or pannus) aware that it may not be differentiated from diffusion of contrast material into the joint fluid (Figure 1).

All patients underwent a detailed clinical assessment including history and thorough rheumatological examination with a complete examination of all joints. As the diagnostic utility and reliability of clinical assessment of the cervical joints are known to be variable and lacking inter-observer agreement [20,21], we defined loss of range of motion as: (1) asymmetry of movement, (2) less than 90 degrees rotation or extension and/or (3) less than 45 degrees of active or passive flexion.

## Results

During the study period 19 JIA patients with cervical symptoms were examined through MRI. 6 patients (3 with seronegative polyarticular JIA, 3 with extended oligoarthritis) were excluded for a missing second (follow-up) MRI examination. The MRIs of these patients all showed joint hyperintensity and enhancement, with malalignment in 2 of them, and none showing ankylosis, erosion or narrowing of the spinal canal at the cranio-cervical junction. The remaining 13 patients (7 females) met the inclusion criteria of clinically and radiologically documented cervical spine arthritis by at least two examinations. Median age at onset of JIA was 6 years (range 1.5 – 12), median age at diagnosis of cervical spine arthritis was 7.7 years (range 1.8 – 14), indicating a median disease duration of 1.7 years until the manifestation of cervical spine arthritis. According to ILAR classification criteria for juvenile idiopathic arthritis [22] patients were diagnosed as extended oligoarthritis (6 patients), seronegative polyarthritis (4 patients), psoriasis arthritis (2 patients), systemic arthritis (1 patient). ANA, HLA-B 27, rheumafactor positivity was seen in 8, 1 and no patients respectively.

At diagnosis of cervical spine arthritis spontaneously reported pain, torticollis, limited range of motion (LROM) were noted in 5, 4, 13 patients respectively. Median duration of treatment with nonsteroidal anti-inflammatory drugs (NSAID), systemic and intraarticular steroids and methotrexate prior to diagnosis of cervical spine arthritis by first MRI patients was 1 year (range 0–11 years). In the course of their disease, patients were treated with the following non-biological agents: NSAIDs (13), glucocorticoids (either methylprednisolon pulses or prednisolon) (13) and methotrexate (13). 12 (93%) patients were treated with the following biologicals: etanercept (11), adalimumab (5), tocilizumab (2). 4 (31%) of the patients had received two different biologicals, and 2 (15%) patients three biologicals. Therapy was changed in 9 of our 13 patients due to first MRI images with seven of them receiving additional biological agents. The median duration of biological therapy was 2.5 years (range: 0.7-8 years). Physiotherapy was conducted in all patients.

At follow-up, after a median disease duration of 2.1 years (range 0.5-8) since the first diagnosis of cervical spine symptoms, clinical assessment revealed fewer patients with spontaneous reported pain and torticollis, although the frequency of loss in range of motion was slightly decreased to 9 patients. 11 patients were still being treated for JIA while 2 patients were in remission and off medication. The median number of extraspinal arthritic joints also had decreased (Table 2).

**Table 2:** Clinical characteristics at diagnosis of cervical spine arthritis and follow-up*

**Clinical characteristic**	**At diagnosis**	**At follow-up**
Spontaneous pain (no. patients)	5	0
Torticollis (no. patients)	4	2
LROM** (no. patients)	13	9
ESR mm/1 hr. median (range)	25 (10–60)	10 (5–35)
Arthritis in extraspinal joints, median number of joints (range)	4 (0–40)	0 (0–35)

*After a median duration of 2.1 years (range 0.5-8 years) of cervical spine arthritis.

**All of them showed asymmetry of cervical spine movement.

Abbr.: *LROM* limited range of motion.

### MRI findings

Tables 3 and 4 show the analysis of the MRI findings: Table 3 indicates the number of involved sites per specific MRI finding. Table 4 indicates the number of patients showing specific MRI findings at the various sites. In most patients multiple sites were involved.

**Table 3:** Number of sites and quality of MRI findings at diagnosis and follow-up (in brackets) in 13 patients

**Qualitiy MRI findings**	**Site**										
	** AA**	** AD**	** AO**	**C2/3**	**C3/4**	**C4/5**	**C5/6**	**C6/7**	**Dens**	**Atlas**	** Sum**
Joint hyperintensity	11 (9)	10 (4)	6 (2)	1 (-)	-	-	-	-	-	-	28 (15)
Joint enhancement	12 (7)	12 (10)	7 (2)	1 (-)	-	-	-	-	-	-	32 (19)
Malalignment	5 (2)	7 (1)	3 (-)	-	-	- (1)	- (1)	- (1)	-	-	15 (6)
Ankylosis	-	-	- (1)	2 (3)	-	- (1)	- (1)	- (1)	-	-	2 (7)
Erosion	-	-	-	-	-	-	-	-	1 (3)	1 (3)	2 (6)
Narrowing of the spinal canal at CCJ									3 (4)		3 (4)

Caption:

Hyperintensity (STIR see Methods) enhancement: pathological joint enhancement after gadolinium.

*AA* atlanto-axial joint.

*AD* atlanto-dental joint.

*AO* atlanto-occipital joint.

*C* facet joint of the cervical spine.

*CCJ* cranio-cervical junction.

**Table 4:** Number of patients and quality of MRI findings at diagnosis and follow-up (in brackets)

**Qualitiy MRI findings**	**Site**															
	**AD + AA + AO**	**AD + AA**	**AA + AO**	**AA**	**AD**	**AD + AA + AQ + C2/3**	**C2/3**	**C2/3 + AO**	**C2/3 + C4-7**	**C3/4**	**C4-7**	**C4-7 + AA**	**Dens + Atlas**	**Dens**	**Atlas**	**Sum**
Joint hyperintensity	5 (1)	3 (3)	0 (1)	2 (4)	1 (0)	1 (0)	-	-	-	-	0/1	-	-	-	-	12 (10)
Joint enhancement	6 (2)	4 (5)	-	1 (0)	1 (3)	1 (0)	-	-	-	0 (1)	-	-	-	-	-	13 (11)
Malalignment	3 (0)	1 (0)	-	1 (1)	3 (1)	-	-	-	-	-	-	0 (1)	-	-	-	8 (3)
Ankylosis	-	-	-	-	-	-	2 (1)	0 (1)	0 (1)	-	-	-	-	-	-	2 (3)
Erosion	-	-	-	-	-	-	-	-	-	-	-	-	0 (2)	1 (1)	1 (1)	2 (4)
Narrowing of the spinal canal at CCJ														3 (4)		3 (4)

Caption see Table 3.

At the time of diagnosis atlanto-axial (AA) and atlanto-dental (AD) joints were most frequently affected as shown by joint hyperintensity and gadolinium enhancement. Altogether 12 (93%) of our patients showed a hyperintense STIR signal and all 13 showed joint enhancement (Table 4).

Erosions were only seen in the dens and atlas (example see Figure 1). Narrowing of cervical spine at the cranio-cervical junction (CCJ) was diagnosed where hypertrophy of the dens was seen (example see Figure 2). Ankylosis (Figure 3) and erosions were seen at 7 sites in 3 patients, and 6 sites in 4 patients at follow-up indicating that these changes may occur at multiple sites in the same patient. Whereas the number of affected patients and sites with joint hyperintensity, enhancement and malalignment decreased at follow-up, ankylosis, erosions and narrowing of the spinal canal at the CCJ increased (Tables 3 and 4).

Figure 3. Two patients with ankylosis. Above: 9-year-old boy (3 mm coronal STIR). Ankylosis with partial fusion of the occipital condyle and atlas on the right with a blurring of the osseous contours on the left. Below: 17-year-old girl with bilateral complete fusion of occipital condyle and atlas (1 mm coronal reconstruction from high-resolution 3D T1 gradient echo post-contrast).

### Associations between clinical and MRI findings

At time of diagnosis all 13 patients showed LROM by asymmetry of cervical movements. 5 patients complained of pain with 3 of them showing ankylosis, erosions or narrowing of CCJ on MRI. At follow-up, 9 (69%) patients showed a LROM (again all realized through the asymmetry of movements) and none complained of pain. 6 (46%) had ankylosis, erosions or narrowing of the spinal canal, all of whom also had a LROM, and two had torticollis. 6 patients were treated with tumor necrosis factor alpha inhibitors including 3 patients who received 2 or more different agents.

## Discussion

MRI allows the direct visualization of synovitis and joint effusion, it shows bone marrow edema and erosions before they become visible on radiographs. Especially in joints that are not accessible to sonography, it is therefore most suitable to detect early signs of arthritis [23]. To date there are no studies documenting the clinical course and MRI follow-up of cervical spine arthritis in JIA. However, this specific manifestation of JIA is important to monitor as it may lead to permanent restriction of cervical spine movements and may considerably impair a patient’s quality of life.

Our study shows that cervical spine arthritis is a serious and persistent manifestation of JIA with severe skeletal sequelae as shown by an increase in number of patients and number of anatomical sites with ankylosis, erosions and narrowing of cranio-cervical junction at follow-up. This is crucial and may include therapeutic implications, particularly because almost all of our patients needed treatment with biologic agents, including most of them requiring therapy intensification due to the diagnosis of cervical spine arthritis. This corroborates the findings of Lee who found that the use of TNF-α inhibitors was an independent risk factor of structural spinal damage [6]. Furthermore, our study shows that MRI specific findings like hyperintensities, enhancement and malalignments allow early diagnosis -as indicated by a median disease duration of 1.7 years after diagnosis of JIA - and that these changes often decrease under therapy while in some patients osseous changes increase. The latter is highlighted by the fact that 6 out of 13 patients (46%) showed ankylosis, erosions or narrowing of the spinal canal at the CCJ after a median disease durationof cervical spine arthritis of 2.1 years. Other studies reported spinal changes only after a median disease duration of 6–24 years using conventional radiography [9-13,24]. Although different imaging techniques, disease characteristics (eg. rheumatoid factor positivity) and duration of disease hamper direct comparison of published results, we think that MRI with its high sensitivity to synovial abnormalities and adjacent bone marrow edema allows diagnosis of cervical spine arthritis earlier than other imaging modalities. This view is supported by the fact that most malalignments were reversible under therapy, probably indicating that these MRI changes are precursors of radiographically documented fixed subluxations. Hence it might be prudent to monitor MRI findings indicative of arthritis in order not to miss transition into irreversible damage like ankylosis, erosions and narrowing of the spinal canal at the cranio-cervical junction.

Our study, in agreement with others, illustrates the importance of LROM as a leading clinical sign of cervical spine arthritis: AA and AD joints were most often affected and these joints account for the major mobility of the cervical spine [11]. Whereas LROM was found in all 13 patients at the time of diagnosis, while only a minority complained of pain indicating that patient history is not a sufficient criterion for cervical spine involvement; a thorough clinical examination is mandatory at every visit. A study based on x-ray findings confirmed a high percentage of asymptomatic patients [9]. While pain improved sufficiently under treatment, LROM was still present in 9 of our patients at follow-up. This persistence of LROM which has been described by others [9,25] is indicative of severe long-term skeletal sequelae.

We believe that patients with osseous changes like ankylosis, erosion or narrowing of the spinal canal at cranio-cervical junction should be advised to restrict excessive or sports activities involving the cervical spine. Narrowing of spinal canal at the cranio-cervical junction caused by hypertrophy of the dens was diagnosed in 4 patients at follow-up. Fortunately none of our patients developed neurological deficit, a poor prognostic factor in rheumatoid arthritis of adulthood: of 21 patients with myelopathy all became bedridden within three years of onset/diagnosis and one third died [26]. In rheumatoid arthritis with symptomatic cervical spine involvement there is also a strong correlation between the development of neurological dysfunction and MRI identification of atlanto-axial spinal canal stenosis [7]. Consequently in cases with neurological symptoms and/or basilar impression, a multidisciplinary approach with experienced MRI investigators and neurosurgeons seems reasonable, and such neurological findings are clearly of utmost importance to anesthetists, where intubation may be necessary for painful interventions such as joint injections or MRI under sedation.

In adult rheumatoid arthritis controversy exists as to whether to perform conventional radiographs or MRI for diagnosis of cervical spine arthritis [7,8]. Studies on imaging procedures in childhood report low sensitivity of radiography: in one study 15 of 29 patients revealed limited cervical spine motion whereas there was radiographic evidence of atlanto-axial subluxation in only 5 [25]. In another study of 20 patients, 65% had soft tissue involvement, pannus formation or erosions on the surface of atlanto-axial joints while only 20% had erosions on plain x-ray views [15]. Given the severity of changes seen in our patients at the onset of disease and the high prevalence of structural cervical spine changes, we would advocate MRI as the procedure of choice for the diagnosis and follow-up of cervical spine involvement in JIA. Only this technique enables a visualization of synovial thickening, increased joint fluid, spinal cord, brainstem and the relationship between occiput, atlas and axis [27]. With projection radiography it would probably not have been possible to monitor the extent of spinal involvement in our patients including those with multiple joint involvement. Moreover MRI reduces radiation exposure, which is especially important in young children. Clearly not all JIA patients need an MR examination of the cervical spine: patients with persistent oligoarthritis do not, or only rarely, develop cervical spine disease [3,11,28-33], however in polyarticular disease cervical spine involvement is frequent both in rheumatoid factor positive and negative patients [9,11]. Hence we would recommend MR imaging in the following situations: (1) signs and symptoms of cervical spine involvement, (2) rheumatoid factor positive and (3) rheumatoid factor negative polyarthritis or extended oligoarthritis with recalcitrant disease necessitating intensive therapy. In cases with narrowing of the spinal canal at cranio-cervical junction or clinically worsening courses, we would recommend control MRI in 6–9 monthly intervals (in milder cases 12 monthly) in order to consider options for treatment intensification.

For reasons of higher specificity as well as comparability of findings, classification criteria for MRI diagnosis of cervical spine involvement are necessary similar to those proposed for radiographic findings [18,19,34]. Regarding the MRI technical procedure, we would like to stress that pathological enhancement revealed additional sites indicating that it is reasonable to apply a contrast medium when performing cervical imaging in JIA patients.

Limitations of our study were the small number of patients and the retrospective study design applying data from medical records, although all patients were examined by the same two pediatric rheumatologists (T.H., J.M.). The lack of exact measurement tools for cervical spine movements in clinical practice might have led to imprecise findings, however all patients with LROM had asymmetry of cervical joint movement in our view, indicating unequivocal findings.

## Conclusion

In conclusion this first MRI based follow-up study shows that cervical spine arthritis can follow a severe course of juvenile arthritis. While malalignments and inflammation sites decreased, osseous changes with erosions, ankylosis and narrowing of the cervical spine junction increased despite minor subjective complaints and treatment with biological agents. Close MRI monitoring of these patients appears to be a sensitive tool for early diagnosis and may help to detect further disease progression and complications.

## Competing interests

The authors declare that they have no competing interests.

## Author’s contribution

All authors contributed to the drafting of the manuscript and approved the final version. TK provided the MRI images.
